# Fruit, vegetable intake and blood pressure trajectories in older age

**DOI:** 10.1038/s41371-019-0189-8

**Published:** 2019-03-06

**Authors:** Denes Stefler, Sofia Malyutina, Yuri Nikitin, Tatiana Nikitenko, Fernando Rodriguez-Artalejo, Anne Peasey, Hynek Pikhart, Severine Sabia, Martin Bobak

**Affiliations:** 1 Department of Epidemiology and Public Health, University College London, London, UK; 2 Research Institute of Internal and Preventive Medicine – Branch of IC&G SB RAS, Novosibirsk, Russia; 3 Department of Preventive Medicine and Public Health, School of Medicine, Universidad Autónoma de Madrid/IdiPaz and CIBERESP, Madrid, Spain; 4 Centre for Research in Epidemiology and Population Health, INSERM U1018, Universite Paris-Saclay, Hopital Paul Brousse, Paris, France

**Keywords:** Risk factors, Hypertension

## Abstract

Diet rich in fruits and vegetables (F&V) is an established protective factor for hypertension, but the available evidence regarding the impact of F&V consumption on age-related blood pressure change is limited. We examined whether systolic (SBP) and diastolic (DBP) blood pressure trajectories are influenced by F&V intakes in an ageing Russian cohort. Dietary data was available for 8997 men and women in the Health, Alcohol and Psychosocial Factors in Eastern Europe prospective cohort study. Blood pressure measurements were taken at three time-points over 12 years of follow-up, during which time the mean age of the sample changed from 58 to 69 years. The relationships between F&V intake and SBP and DBP were assessed using mixed-effect multilevel models. In the multivariable adjusted models, fruit intake was inversely related to both systolic and diastolic blood pressure at baseline (mean SBP and DBP was 3.5 mmHg and 1.4 mm Hg lower in the highest compared to the lowest intake tertiles, respectively (both *p* values < 0.001)). However, it was not associated with blood pressure change over time (difference in annual SBP and DBP change was 0.11 mmHg (*p* value = 0.138) and 0.01 mmHg (*p* value = 0.894), respectively). We found no significant link between vegetable intake and blood pressure, neither cross-sectionally nor longitudinally. In addition to the association with diet, we observed increasing SBP and mostly steady DBP over age, with deceleration and downward turn after the ages of 55–59 years. On the whole, this analysis found no consistent association between F&V intake and trajectories of blood pressure in older age.

## Introduction

Blood pressure changes with age. It gradually increases during most part of the adulthood, reaching the highest values around the mid-60 s or early 70 s; after this age, as most studies suggest, it stays steady or turns into slow decline [1–4]. Although these trends in blood pressure over the life course have been described by several longitudinal analyses, few previous studies examined the factors which influence this trajectory in adult and elderly individuals.

Strong evidence, supported by large observational and experimental studies, indicate that fruit and vegetable (F&V) consumption is inversely related to blood pressure [5–7]. In fact, the reduction in cardiovascular disease (CVD) and overall mortality risk associated with F&V intake is likely to be, at least partially, the result of its blood pressure lowering effect. However, the available evidence regarding the effect of F&V intake on age-related blood pressure change is limited, and mainly supported by studies with relatively short (5–7 years) follow-up [8–10].

Cardiovascular morbidity and mortality rates in Eastern European countries have been historically high, and hypertension among adults is also more prevalent here than in most Western European states [11–13]. This disease pattern coincides with high prevalence of poor diet, including low fruit intakes, particularly in Russia [14, 15].

Using longitudinal data from the Russian arm of the Health, Alcohol and Psychosocial Factors in Eastern Europe (HAPIEE) study, the main aim of this analysis was to examine whether systolic and diastolic blood pressure trajectories are influenced by fruit or vegetable intakes in adult and elderly individuals over a follow-up of 12 years.

## Methods

### Study population

HAPIEE study is a multi-centre prospective cohort which was established to investigate non-communicable chronic diseases, primarily CVD, and their risk factors in four Eastern European countries [16]. The Russian arm of the study recruited participants between the ages of 45 and 70 years in the city of Novosibirsk, selected from electoral list using age and sex-stratified random sampling method (response rate at baseline: 61%). Baseline examination took place in 2003–2005 (wave 1) which was followed by two subsequent waves in 2006–2008 (wave 2) and 2015–2017 (wave 3). Full sample sizes in the three waves were 9360, 6182 and 3789, respectively.

All participants signed informed consent and study protocols were approved by ethical committees at University College London and Institute of Internal and Preventive Medicine in Novosibirsk.

### Data collection

Nutritional data collection procedures in the HAPIEE study have been described previously in details [17, 18]. Briefly, diet at baseline (wave 1) was assessed using a semi-quantitative food frequency questionnaire (FFQ) with 147 items. Participants indicated how frequently they consumed a portion of a specific food item on a 9-point scale, and daily intake was calculated by multiplying the number of portions with the average portion size. The European Food Safety Authority’s FoodEx 2 food classification and description system was used to categorise food items into fruits (21 items) and vegetables (24 items) [19]. In a previous analysis, validity of the FFQ regarding F&V consumption was examined using biomarkers [20]. The correlations between fruit, vegetable intakes and vitamin C and beta-carotene plasma concentrations were moderate (*r* = 0.26), similarly to many other large-scale studies [21], which suggest acceptable validity of the FFQ.

Systolic (SBP) and diastolic blood pressure (DBP) measurements were taken in all three waves of the study by trained nurses following a standard protocol using an Omron M5-I digital sphygmomanometer. Subjects were in a sitting position after 5 min of quiet rest. The measurements were taken on the right arm which was bent in 45-degree at the elbow and supported by a flat surface (table). Three measurements were taken with 2 min intervals between them. The means of the second and third measurement of systolic and diastolic blood pressure values were used in the analyses.

Baseline measurements of self-reported smoking (never-; ex-; current-smoker), frequency of alcohol consumption (several times a week; less than once a week; never), leisure time physical activity (<3 MET-hours per day; 3–10 MET-hours per day; >10 MET-hours per day), marital status (living alone; living with partner), education (less than primary; vocational; secondary; university) and energy intake (from FFQ) were used as covariates in the statistical analyses. Information on antihypertensive medication use (yes; no) was assessed at all three data collection waves.

### Analytical sample size and attrition

In line with recommendations for nutritional epidemiological analyses [22], we excluded participants with extreme reported energy intake (more than 4500 kcal/day or less than 500 kcal/day for women; more than 5000 kcal/day or less than 800 kcal/day for men), those with more than 10% missing FFQ answers and individuals who indicated that the FFQ was not representative of their diet. Consequently, the number of participants who were included in the analysis in waves 1, 2 and 3 was 8997, 5966 (66.3% of wave 1) and 3667 (40.8%), respectively (Table S1 in supplementary material).

The attrition of the sample in waves 2 and 3 was due to deaths (*n* = 1756) and losses of follow-up for other reasons (*n* = 3574). Compared to those who remained in the study, individuals lost to follow-up were older, more likely to be males, smokers and with lower levels of education. Their SBP and DBP were also higher at baseline (table S2 in supplementary material).

### Statistical analysis

Participants were categorised into tertiles based on their fruit and vegetable intakes. These categorical variables were then used as our main exposures. In order to model SBP and DBP trajectories across the three measurement waves, over an average of 12 years follow-up, mixed-effect multilevel models were used [23]. This statistical method is particularly recommended for growth curves when time intervals between measurement waves vary considerably across individuals, and its flexibility in dealing with missing data is a further advantage [23, 24].

Both the intercept and slope were fitted as random effects, allowing individual differences in blood pressure at baseline and rate of change. The models were adjusted for the covariates, time since baseline (wave 1) and interaction terms between each covariate and time.

The obtained coefficients for F&V intake tertiles *without* interaction with time provided information on their cross-sectional relationship with blood pressure (intercept), whereas the coefficients *with* interaction between F&V intake and time indicated the respective longitudinal association (change in SBP or DBP per one year of follow-up time) (slope).

Models were first adjusted for sex, age (centered at 58 years), antihypertensive medication use (as time-varying covariate) and energy intake (centered at 10.6 MJ/day), corresponding to model 1. In model 2, they were further adjusted for education, marital status, smoking, alcohol intake, leisure time physical activity and fruit or vegetable intake.

In order to ease interpretation of the results, fully adjusted blood pressure trajectories in the different fruit or vegetable intake tertiles were also plotted separately by 5-year birth cohorts using the ‘margins’ command in Stata (StataCorp, TX, US).

Statistical analyses were carried out using the MLwiN v3.01 software which was accessed through Stata v13.1 with the help of the ‘runmlwin’ command [25].

### Data and code availability

Availability of data from the HAPIEE study is restricted due to legal reasons. Further information and access, including on availability of statistical codes, can be requested by contacting the principle investigator, Professor Martin Bobak (m.bobak@ucl.ac.uk) who will seek approval by the HAPIEE Study Steering Committee and the Research Ethics Committee at UCL and participating centres.

## Results

Table 1 shows the characteristics of the sample across fruit and vegetable intake tertiles at baseline. Average consumption of fruits (mean (SD): 152.5 (168.4) g/day) was considerably lower than vegetables (mean (SD): 266.6 (169.5) g/day). Most demographic, socio-economic and lifestyle characteristics were significantly related to both fruit and vegetable intakes. Females, non-smokers, higher educated participants and those who take antihypertensive medications or live with partners were more likely to eat higher amounts of fruits and vegetables. On the other hand, alcohol intake was associated only with fruit intake, and we also found an unexpected inverse association between physical activity and vegetable consumption.

**Table 1:** Characteristics of the sample at baseline by fruit and vegetable intake tertiles

Covariates	Fruit intake tertiles							Vegetable intake tertiles						
	T1 (lowest)		T2		T3 (highest)			T1 (lowest)		T2		T3 (highest)		
	Mean	(SD)	Mean	(SD)	Mean	(SD)	*p* value	Mean	(SD)	Mean	(SD)	Mean	(SD)	*p* value
Age (years)	59.4	(6.9)	57.9	(7.0)	56.9	(6.7)	<0.001	58.1	(7.0)	57.7	(6.9)	58.5	(7.0)	<0.001
Daily energy intake (MJ)	9.6	(2.9)	10.6	(3.1)	11.6	(3.2)	<0.001	9.7	(3.0)	10.6	(3.0)	11.5	(3.2)	<0.001
Body Mass Index (kg/m2)	28.1	(5.6)	28.9	(5.4)	29.0	(5.4)	<0.001	28.2	(5.4)	28.7	(5.5)	28.8	(5.5)	<0.001
Fruit intake (g/day)	35.1	(20.2)	108.5	(25.3)	314.2	(205.3)	<0.001	108.5	(112.4)	136.0	(125.2)	213.1	(225.5)	<0.001
Vegetable intake (g/day)	208.0	(109.2)	253.9	(127.0)	337.8	(222.5)	<0.001	135.1	(40.9)	226.0	(26.9)	438.6	(187.8)	<0.001
Mediterranean diet score^a^	5.5	(1.6)	5.5	(1.6)	5.5	(1.6)	0.562	5.3	(1.6)	5.6	(1.7)	5.7	(1.6)	<0.001
	%		%		%		*p*-value	%		%		%		*p*-value
Gender: females	46.3		54.3		63.9		<0.001	51.9		55.1		57.6		<0.001
Antihypertensive medication: yes	28.8		31.7		35.6		<0.001	30.1		32.0		34.0		0.005
Smoking: current smoker	35.5		27.3		21.5		<0.001	30.5		27.6		26.2		<0.001
Alcohol intake: several times a week	27.1		23.6		22.3		<0.001	24.3		25.1		23.6		0.762
Leisure time physical activity: > 10 MET-h/d	33.4		35.7		39.2		<0.001	40.7		33.5		34.2		<0.001
Education: university	21.3		29.3		36.4		<0.001	27.6		29.5		29.8		<0.001
Marital status: lives with partner	68.4		73.8		74.3		<0.001	69.8		73.9		72.9		0.001

*P* values were calculated with ANOVA for continuous variables and with Chi-square test for categorical variables

^a^Without the fruit, vegetable and alcohol component [27]

Cross-sectional (intercept) and longitudinal (slope) associations between fruit and vegetable intakes and SBP and DBP are presented in Table 2. At baseline, individuals with higher fruit intakes had significantly lower blood pressure. For example, in the multivariable adjusted models, mean SBP and DBP in the highest fruit intake tertile were 3.2 mmHg and 1.1 mmHg lower than in the lowest tertile, respectively (both *p* < 0.001). However, the cross-sectional associations with vegetable intake were not statistically significant (*p* = 0.204 for SBP and *p* = 743 for DBP). Longitudinally, the coefficients indicated mostly non-significant associations and inconsistent trends across the tertiles for both fruit and vegetable intakes.

**Table 2:** Cross-sectional and longitudinal relationship between fruit and vegetable intakes and SBP and DBP

Outcome	Food group	Category	Blood pressure at baseline (mmHg) (Cross-sectional association)						Blood pressure change per 1-year of follow-up (mmHg) (Longitudinal association)					
			Model 1			Model 2			Model 1			Model 2		
			Mean	(95%CI)	*p* value	Mean	(95%CI)	*p* value	Mean	(95%CI)	*p* value	Mean	(95%CI)	*p* value
SBP	FRUIT	1st tertile	141.4	(140.3, 142.4)	ref.	143.0	(140.4, 145.5)	ref.	0.05	(−0.12, 0.21)	ref.	0.16	(−0.20, 0.53)	ref.
		2nd tertile	139.8	(138.6, 141.0)	0.007	141.5	(138.9, 144.2)	0.013	0.09	(−0.07, 0.26)	0.481	0.22	(−0.15, 0.59)	0.422
		3rd tertile	137.9	(136.6, 139.2)	<0.001	139.8	(137.1, 142.5)	<0.001	0.16	(−0.02, 0.33)	0.124	0.27	(−0.10, 0.65)	0.138
	VEGETABLE	1st tertile	141.4	(140.3, 142.4)	ref.	143.0	(140.4, 145.5)	ref.	0.05	(−0.12, 0.21)	ref.	0.16	(−0.20, 0.53)	ref.
		2nd tertile	142.0	(140.9, 143.1)	0.254	143.3	(140.7, 145.9)	0.537	−0.10	(−0.27, 0.07)	0.029	0.04	(−0.33, 0.41)	0.075
		3rd tertile	142.3	(141.1, 143.6)	0.110	143.7	(141.1, 146.3)	0.204	0.03	(−0.15, 0.21)	0.846	0.17	(−0.20, 0.54)	0.894
DBP	FRUIT	1st tertile	89.5	(88.9, 90.1)	ref.	89.2	(87.8, 90.7)	ref.	−0.01	(−0.10, 0.08)	ref.	0.14	(−0.06, 0.35)	ref.
		2nd tertile	89.0	(88.3, 89.7)	0.147	88.7	(87.2, 90.2)	0.141	−0.09	(−0.18, 0.00)	0.031	0.07	(−0.14, 0.27)	0.051
		3rd tertile	88.1	(87.4, 88.8)	<0.001	87.9	(86.4, 89.4)	<0.001	−0.02	(−0.12, 0.08)	0.763	0.13	(−0.08, 0.34)	0.906
	VEGETABLE	1st tertile	89.5	(88.9, 90.1)	ref.	89.2	(87.8, 90.7)	ref.	−0.01	(−0.10, 0.08)	ref.	0.14	(−0.06, 0.35)	ref.
		2nd tertile	89.8	(89.2, 90.5)	0.271	89.4	(88.0, 90.9)	0.562	−0.03	(−0.13, 0.06)	0.516	0.13	(−0.07, 0.33)	0.762
		3rd tertile	89.7	(89.0, 90.4)	0.534	89.3	(87.9, 90.8)	0.743	0.04	(−0.06, 0.14)	0.251	0.20	(−0.01, 0.40)	0.178

Model 1: adjusted for age, sex, energy intake and antihypertensive medication

Model 2: adjusted for all variables in model 1, plus education, marital status, smoking, frequency of alcohol intake and leisure time physical activity. Fruit and vegetable intakes were also adjusted for each other. (All covariates were included in the models both with and without interaction with time. Age and energy intake were centered at the mean – (58 years, 10.6MJ))

**Fig. 1:**
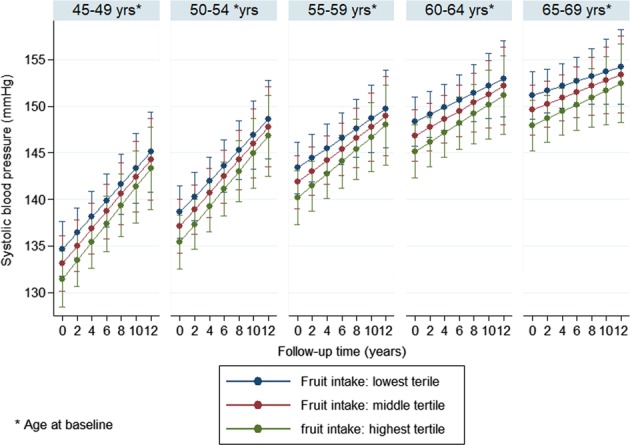
Systolic blood pressure trajectories by fruit intake tertiles (separately by 5-year birth cohorts)

**Fig. 2:**
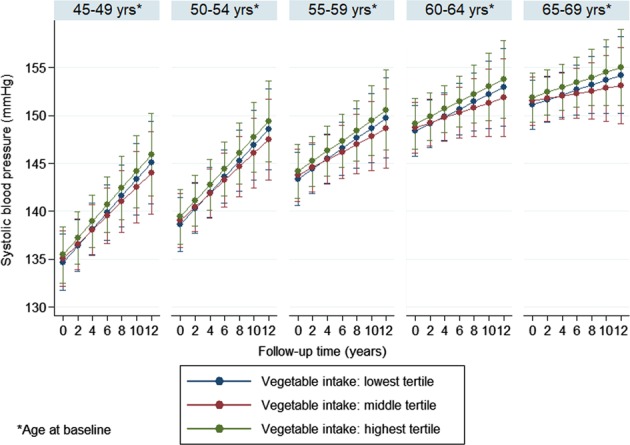
Systolic blood pressure trajectories by vegetable intake tertiles (separately by 5-year birth cohorts)

**Fig. 3:**
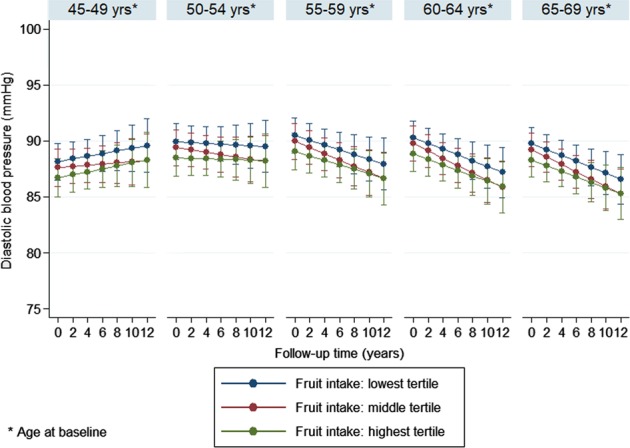
Diastolic blood pressure trajectories by fruit intake tertiles (separately by 5-year birth cohorts)

**Fig. 4:**
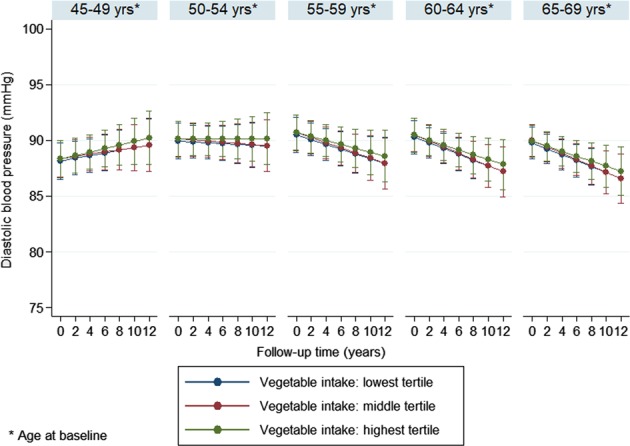
Diastolic blood pressure trajectories by vegetable intake tertiles (separately by 5-year birth cohorts)

Multivariable adjusted (model 2) trajectories, separately by 5-year birth cohorts, are shown in Figs. 1–4. The results suggest that SBP increased in participants over time, which trend was steeper in the younger age groups compared to the older ones. The figures also indicate that, for fruit intake, the increase in SBP was somewhat steeper in the highest tertile compared to the lowest and middle groups, while regarding vegetable consumption, the middle tertile showed considerably flatter trend than the other two groups. In contrast to SBP, DBP remained mostly steady over time in the two youngest age groups, and a downward trend could be observed in the older participants. The steepest decline in DBP was observed in the middle tertile regarding fruit intake, but no substantial difference in the speed of change was found across vegetable intake tertiles.

Although multilevel modelling can adequately deal with missing data under the missing at random assumption [23, 24], we also repeated our main analysis on complete cases with available data in all three waves (see table S3, figure S1 and S2 in supplementary material), as well as after imputing the missing data with multiple random imputation procedure in all participants included in the main analysis (see table S4 in supplementary material) [26]. The results of these sensitivity analyses were not substantially different from our main findings regarding the differences in trajectories across F&V intake tertiles.

In further sensitivity analyses, we assessed the associations only among those participants who took no antihypertensive medications (see table S5, figure S3 and S4 in supplementary material). The results indicated that the increase of SBP and DBP over time was steeper and the downward change of blood pressure trends in the older age groups was less pronounced in those who took no blood pressure medication. Nonetheless, for the comparison across F&V intakes, these analyses provided similar results to the main findings.

Further adjustment for BMI or a modified Mediterranean diet score [27] did not materially change the observed trajectories and their associations with F&V intake (Tables S6 and S7 in supplementary material). The results also remained similar when participants with previous CVD in their medical history were excluded from the analysis (Table S8 in Supplementary material).

## Discussion

### Main findings

This longitudinal study on blood pressure trajectories in an ageing Russian cohort showed that fruit intake was inversely related to both systolic and diastolic blood pressure at baseline, but it had no clear impact on the age-related blood pressure change over 12 years of follow-up. Furthermore, we found no obvious link between vegetable intake and blood pressure, neither cross-sectionally nor longitudinally. In addition to the association with diet, we also observed increasing SBP and mostly steady DBP over age, however, these trends changed when participants reached their late 50 s/early 60 s and became less steep for SBP and turned into a decline for DBP.

### Interpretation

Cross-sectional results of our analysis support previous data for the beneficial health effects of fruits, but less so for vegetables [6]. Our previous analysis and food availability data from the UN’s Food and Agricultural Organisation suggest that vegetable intake in Russia is relatively high [15, 28]. However, they are usually consumed in a preserved or cooked form, rather than raw, for example, as salads [29], and it is possible that added salt during cooking and preservation procedures might counteract with the vegetables’ inherent blood pressure lowering effect. Although accurate measurement of salt intake is problematic, further studies which examine the association between vegetable intake and blood pressure in Russian or other Eastern European samples, with appropriate adjustment for salt intake, would be necessary to clarify this question.

The specific types of fruit and vegetables might be also important. For example, among fruits, berries (raspberry, strawberry, red currant, etc.) are particularly popular in Russia. This is mainly due to the climatic conditions as these are the fruits which can grow relatively well in continental and subarctic climates typical to large part of the country, including the Novosibirsk region in Western Siberia. There is some evidence which suggest that berries may be less effective in the prevention of CHD and stroke than many other types of fruit [30]. Therefore, this characteristic of the examined population can also affect our findings regarding the link between fruit intake and blood pressure

There are a number of potential explanations for the lack of clear association between F&V intake and longitudinal blood pressure change. Firstly, it is possible that measurement error of dietary intakes reduced the capacity of the data to identify actually existing associations. However, the adequate validity of the FFQ in relation to biomarkers [20], the fact that the cross-sectional results for fruit intake pointed to the expected direction and that previous survival analysis in the HAPIEE cohort showed inverse associations between F&V intake and mortality outcomes [18] makes this possibility less likely. Secondly, other unmeasured factors, such as the above mentioned salt intake, may have a larger impact on the blood pressure trends, and could have masked the association with F&V intake. However, we controlled our models for the most likely confounders, including both socio-economic and lifestyle factors, reducing considerably the room for residual confounding. Thirdly, we assessed dietary intake only at baseline and it is possible that participants changed their F&V intake habits during follow-up, which could have led to misclassification and reduction in the effect size. Future studies are recommended to apply repeated dietary assessments in order to maximise the probability of positive findings.

The inverse relationship with fruit intake at baseline together with the parallel blood pressure trends across intake groups over time also mean that the beneficial effects of fruit consumption were maintained during the observational period. This suggests that eating adequate amount of fruits throughout the lifecourse do have an impact on blood pressure in older age even if its effect on the age-related change itself is limited.

Previous analyses suggest a constant increase in blood pressure from early to late adulthood with the steepest rise between 40 and 55 years [2, 4]. After the age of 65, several studies indicate steady or decreasing trends, which may be partially explained by antihypertensive drug treatments, but other factors probably also play a role [2, 31]. Our findings on the examined Russian cohort correspond well with these earlier studies which were carried out using data from Western European and North American population samples. The current results also provide further evidence that antihypertensive medication use in older age or survival bias cannot entirely explain the deceleration of blood pressure change after the age of 65. The steeper increase in SBP compared to DBP in the younger age groups is also consistent with previous findings [32].

### Limitations and strengths

In addition to the potential impacts of measurement bias and residual confounding on the results, which were mentioned above, we also acknowledge the fact that, due to the moderate response rate and restrictions during sampling (only urban individuals were included), our sample is not fully representative to the Russian population as a whole. It is likely that the current cohort is healthier than the general population [16]. However, this should not affect the internal validity of the findings.

On the other hand, our study has important strengths as well. This is one of the first longitudinal analyses which examined the impact of dietary factors on blood pressure trajectories in adult/elderly individuals over more than ten years of follow-up. In addition, we carried out the analysis using data from an Eastern European cohort, representing a population which often lacks good quality epidemiological data.

## Conclusion

High blood pressure is one of the most important modifiable risk factors for CVD and overall mortality. Investigating the lifestyle habits which can influence its trajectories in older age has a clear practical importance. Despite our inconsistent findings, F&V intake remains a prime behaviour to reduce blood pressure and further research in this topic is strongly warranted.

### What is known about the topic

- Systolic and diastolic blood pressure changes with age with a mostly increasing trend in mid and late adulthood.
- High fruit and vegetable intake is an established protective factor for hypertension.

### What this study adds

- Using data from a Russian population sample, this study confirms that blood pressure increases during older age, but the trend changes in the 60 s, after which age it stays steady or potentially turns into a decline.
- Fruit intake is inversely associated with systolic and diastolic blood pressure, but the impact of fruit or vegetable intake on age-related blood pressure change remains unclear.

## Supplementary information

Supplemental material
