# Supplementary material for: Fruit, vegetable intake and blood pressure trajectories in older age
Source: J Hum Hypertens. 2019 Mar 6;33(9):671–8. doi: 10.1038/s41371-019-0189-8 (PMC6760602; doi:10.1038/s41371-019-0189-8)

**Table S1.** Main characteristics of participants in the Russian arm of the HAPIEE study across three waves of data collection

|  | **Wave 1**  (2002-2005) | |  | **Wave 2**  (2006-2008) | |  | **Wave 3**  (2015-2017) | |
| --- | --- | --- | --- | --- | --- | --- | --- | --- |
| Characteristics | n=8997 | |  | n=5966 | |  | n=3667 | |
|  | mean | (sd) |  | mean | (sd) |  | mean | (sd) |
| Follow-up time since Wave 1 (years) | 0.0 |  |  | 3.0 | (0.7) |  | 12.4 | (0.7) |
| Age (years) | 58.1 | (7.0) |  | 61.3 | (6.9) |  | 69.3 | (6.8) |
| Systolic Blood Pressure (mmHg) | 142.7 | (24.6) |  | 146.1 | (24.1) |  | 144.2 | (21.0) |
| Diastolic Blood Pressure (mmHg) | 90.0 | (13.3) |  | 90.9 | (13.3) |  | 83.1 | (11.4) |
|  | % |  |  | % |  |  | % |  |
| Gender: Females | 54.9 |  |  | 56.5 |  |  | 61.9 |  |
| Antihypertensive medication use | 32.1 |  |  | 42.6 |  |  | 62.2 |  |

**Table S2.** Difference at baseline between individuals who were lost during follow–up and those who remained in the study until wave 3

|  | Lost during follow-up  (n=5330) | |  | Not lost during follow-up  (n=3667) | |  |
| --- | --- | --- | --- | --- | --- | --- |
|  | *mean* | *(sd)* |  | *mean* | *(sd)* | p-value* |
| Age (years) | 58.9 | (7.0) |  | 57.0 | (6.7) | <0.001 |
| Systolic Blood Pressure (mmHg) | 145.0 | (25.3) |  | 139.4 | (23.3) | <0.001 |
| Diastolic Blood Pressure (mmHg) | 90.8 | (13.5) |  | 88.8 | (12.9) | <0.001 |
| Fruit intake (g/day) | 143.0 | (164.4) |  | 166.4 | (173.1) | <0.001 |
| Vegetable intake (g/day)* | 261.0 | (162.0) |  | 274.7 | (179.6) | <0.001 |
| Energy intake (MJ) | 10.5 | (3.2) |  | 10.7 | (3.1) | <0.001 |
| Body Mass Index (kg/m2) | 28.6 | (5.7) |  | 28.4 | (5.0) | 0.076 |
|  | *%* |  |  | *%* |  |  |
| Gender: Females | 50.0 |  |  | 61.9 |  | <0.001 |
| Antihypertensive medication: yes | 33.5 |  |  | 29.9 |  | <0.001 |
| Smoking: current smoker | 32.9 |  |  | 21.2 |  | <0.001 |
| Alcohol: every day or several times a week | 25.2 |  |  | 23.1 |  | <0.001 |
| Leisure time physical activity: >10 MET-h/d | 33.0 |  |  | 40.7 |  | <0.001 |
| Education: University | 25.2 |  |  | 34.4 |  | <0.001 |
| Marital status: lives with partner | 71.2 |  |  | 73.3 |  | 0.010 |

*P-values were calculated with t-test for continuous variables and chi-square test for categorical variables

**Table S3.** Cross-sectional and longitudinal relationship between fruit and vegetable intakes and SBP and DBP among individuals who participated in all three waves of the study **(complete case analysis) (n=3132)**

| Outcome | Food group | Category | **Blood pressure at baseline (mmHg)**  (Cross-sectional association) | | |  | **Blood pressure change per 1-year of follow up (mmHg)**  (Longitudinal association) | | |
| --- | --- | --- | --- | --- | --- | --- | --- | --- | --- |
|  |  |  | **mean** | (95% CI) | p-value |  | **mean** | (95% CI) | p-value |
| **SBP** | **FRUIT** | 1st tertile | **138.6** | (133.8, 143.4) | ref. |  | **0.23** | (-0.22, 0.69) | ref. |
|  |  | 2nd tertile | **135.9** | (131.0, 140.8) | 0.004 |  | **0.35** | (-0.11, 0.81) | 0.173 |
|  |  | 3rd tertile | **135.3** | (130.3, 140.2) | 0.001 |  | **0.38** | (-0.09, 0.84) | 0.108 |
|  | **VEGETABLE** | 1st tertile | **138.6** | (133.8, 143.4) | ref. |  | **0.23** | (-0.22, 0.69) | ref. |
|  |  | 2nd tertile | **139.3** | (134.5, 144.0) | 0.477 |  | **0.09** | (-0.36, 0.54) | 0.074 |
|  |  | 3rd tertile | **139.8** | (135.1, 144.6) | 0.208 |  | **0.23** | (-0.22, 0.68) | 0.975 |
| **DBP** | **FRUIT** | 1st tertile | **86.8** | (84.1, 89.6) | ref. |  | **0.12** | (-0.13, 0.38) | ref. |
|  |  | 2nd tertile | **85.8** | (83.0, 88.6) |  |  | **0.09** | (-0.17, 0.35) | 0.483 |
|  |  | 3rd tertile | **85.4** | (82.5, 88.2) | 0.010 |  | **0.14** | (-0.12, 0.40) | 0.690 |
|  | **VEGETABLE** | 1st tertile | **86.8** | (84.1, 89.6) | ref. |  | **0.12** | (-0.13, 0.38) | ref. |
|  |  | 2nd tertile | **87.0** | (84.3, 89.7) | 0.751 |  | **0.09** | (-0.17, 0.34) | 0.475 |
|  |  | 3rd tertile | **87.2** | (84.5, 89.9) | 0.515 |  | **0.15** | (-0.10, 0.40) | 0.542 |

All coefficients were adjusted for age, sex, energy intake, education, marital status, smoking, frequency of alcohol intake and leisure time physical activity. Fruit and vegetable intakes were also adjusted for each other. (All covariates were included in the models both with and without interaction with time. Age and energy intake were centered at the mean – (58yrs, 10.6MJ))

**Table S4.** Cross-sectional and longitudinal relationship between fruit and vegetable intakes and SBP and DBP among individuals **after multiple random imputation* of missing data**

| Outcome | Food group | Category | **Blood pressure at baseline (mmHg)**  (Cross-sectional association) | | |  | **Blood pressure change per 1-year of follow up (mmHg)**  (Longitudinal association) | | |
| --- | --- | --- | --- | --- | --- | --- | --- | --- | --- |
|  |  |  | **mean** | (95% CI) | p-value |  | **mean** | (95% CI) | p-value |
| **SBP** | **FRUIT** | 1st tertile | **143.5** | (140.8, 146.2) | ref. |  | **-0.21** | (-0.68, 0.26) | ref. |
|  |  | 2nd tertile | **142.3** | (139.4, 145.2) | 0.049 |  | **-0.13** | (-0.58, 0.32) | 0.317 |
|  |  | 3rd tertile | **140.6** | (137.7, 143.5) | <0.001 |  | **-0.11** | (-0.58, 0.36) | 0.244 |
|  | **VEGETABLE** | 1st tertile | **143.5** | (140.8, 146.2) | ref. |  | **-0.21** | (-0.68, 0.26) | ref. |
|  |  | 2nd tertile | **144.0** | (141.3, 146.8) | 0.371 |  | **-0.31** | (-0.78, 0.16) | 0.169 |
|  |  | 3rd tertile | **144.8** | (142.0, 147.6) | 0.043 |  | **-0.24** | (-0.73, 0.26) | 0.707 |
| **DBP** | **FRUIT** | 1st tertile | **89.7** | (88.1, 91.2) | ref. |  | **-0.06** | (-0.32, 0.20) | ref. |
|  |  | 2nd tertile | **89.2** | (87.6, 90.8) | 0.189 |  | **-0.11** | (-0.36, 0.13) | 0.245 |
|  |  | 3rd tertile | **88.4** | (86.8, 90.0) | 0.001 |  | **-0.06** | (-0.32, 0.20) | 0.992 |
|  | **VEGETABLE** | 1st tertile | **89.7** | (88.1, 91.2) | ref. |  | **-0.06** | (-0.32, 0.20) | ref. |
|  |  | 2nd tertile | **90.0** | (88.5, 91.5) | 0.320 |  | **-0.07** | (-0.32, 0.17) | 0.764 |
|  |  | 3rd tertile | **90.1** | (88.6, 91.7) | 0.198 |  | **-0.04** | (-0.30, 0.21) | 0.630 |

All coefficients were adjusted for age, sex, energy intake, antihypertensive medication use, education, marital status, smoking, frequency of alcohol intake, and leisure time physical activity. Fruit and vegetable intakes were also adjusted for each other. (All covariates were included in the models both with and without interaction with time. Age and energy intake were centered at the mean – (58yrs, 10.6MJ))

* Multiple chained imputation procedure was used in Stata v 13.1. Fifty imputed datasets were created, and in addition to all variables which the associations were adjusted for (listed above), data on all-cause mortality, CVD in history and BMI were also included in the imputation procedure.

**Table S5.** Cross-sectional and longitudinal relationship between fruit and vegetable intakes and SBP and DBP **among those participants who took no antihypertensive medications**

| Outcome | Food group | Category | **Blood pressure at baseline (mmHg)**  (Cross-sectional association) | | |  | **Blood pressure change per 1-year of follow up (mmHg)**  (Longitudinal association) | | |
| --- | --- | --- | --- | --- | --- | --- | --- | --- | --- |
|  |  |  | **mean** | (95% CI) | p-value |  | **mean** | (95% CI) | p-value |
| **SBP** | **FRUIT** | 1st tertile | **142.1** | (139.1, 145.2) | ref. |  | **0.87** | (0.34, 1.40) | ref. |
|  |  | 2nd tertile | **140.6** | (137.5, 143.8) | 0.025 |  | **0.92** | (0.38, 1.46) | 0.595 |
|  |  | 3rd tertile | **139.4** | (136.2, 142.6) | <0.001 |  | **0.92** | (0.37, 1.47) | 0.649 |
|  | **VEGETABLE** | 1st tertile | **142.1** | (139.1, 145.2) | ref. |  | **0.87** | (0.34, 1.40) | ref. |
|  |  | 2nd tertile | **142.2** | (139.2, 145.2) | 0.925 |  | **0.77** | (0.23, 1.30) | 0.270 |
|  |  | 3rd tertile | **141.9** | (138.8, 144.9) | 0.736 |  | **0.99** | (0.45, 1.52) | 0.247 |
| **DBP** | **FRUIT** | 1st tertile | **88.7** | (86.9, 90.5) | ref. |  | **0.49** | (0.19, 0.79) | ref. |
|  |  | 2nd tertile | **88.2** | (86.4, 90.0) | 0.187 |  | **0.43** | (0.12, 0.74) | 0.259 |
|  |  | 3rd tertile | **87.6** | (85.7, 89.4) | 0.007 |  | **0.52** | (0.21, 0.83) | 0.674 |
|  | **VEGETABLE** | 1st tertile | **88.7** | (86.9, 90.5) | ref. |  | **0.49** | (0.19, 0.79) | ref. |
|  |  | 2nd tertile | **88.8** | (87.0, 90.5) | 0.865 |  | **0.48** | (0.17, 0.78) | 0.787 |
|  |  | 3rd tertile | **88.5** | (86.8, 90.3) | 0.672 |  | **0.59** | (0.29, 0.89) | 0.091 |

All coefficients were adjusted for age, sex, energy intake, education, marital status, smoking, frequency of alcohol intake and leisure time physical activity. Fruit and vegetable intakes were also adjusted for each other. (All covariates were included in the models both with and without interaction with time. Age and energy intake were centered at the mean – (58yrs, 10.6MJ))

**Table S6.** Cross-sectional and longitudinal relationship between fruit and vegetable intakes and SBP and DBP **after further adjustment for BMI**

| Outcome | Food group | Category | **Blood pressure at baseline (mmHg)**  (Cross-sectional association) | | |  | **Blood pressure change per 1-year of follow up (mmHg)**  (Longitudinal association) | | |
| --- | --- | --- | --- | --- | --- | --- | --- | --- | --- |
|  |  |  | **mean** | (95% CI) | p-value |  | **mean** | (95% CI) | p-value |
| **SBP** | **FRUIT** | 1st tertile | **144.5** | (141.9, 147.0) | ref. |  | **0.12** | (-0.25, 0.49) | ref. |
|  |  | 2nd tertile | **142.8** | (140.2, 145.5) | 0.004 |  | **0.18** | (-0.19, 0.55) | 0.408 |
|  |  | 3rd tertile | **141.1** | (138.4, 143.8) | <0.001 |  | **0.23** | (-0.15, 0.61) | 0.132 |
|  | **VEGETABLE** | 1st tertile | **144.5** | (141.9, 147.0) | ref. |  | **0.12** | (-0.25, 0.49) | ref. |
|  |  | 2nd tertile | **144.6** | (142.1, 147.1) | 0.810 |  | **0.00** | (-0.36, 0.37) | 0.086 |
|  |  | 3rd tertile | **145.0** | (142.5, 147.6) | 0.330 |  | **0.14** | (-0.23, 0.51) | 0.792 |
| **DBP** | **FRUIT** | 1st tertile | **90.4** | (89.0, 91.8) | ref. |  | **0.10** | (-0.11, 0.30) | ref. |
|  |  | 2nd tertile | **89.7** | (88.3, 91.2) | 0.043 |  | **0.03** | (-0.18, 0.23) | 0.068 |
|  |  | 3rd tertile | **88.9** | (87.4, 90.4) | <0.001 |  | **0.09** | (-0.12, 0.30) | 0.911 |
|  | **VEGETABLE** | 1st tertile | **90.4** | (89.0, 91.8) | ref. |  | **0.10** | (-0.11, 0.30) | ref. |
|  |  | 2nd tertile | **90.4** | (89.0, 91.8) | 0.969 |  | **0.09** | (-0.11, 0.30) | 0.917 |
|  |  | 3rd tertile | **90.4** | (88.9, 91.8) | 0.907 |  | **0.16** | (-0.05, 0.36) | 0.121 |

All coefficients were adjusted for age, sex, energy intake, antihypertensive medication, education, marital status, smoking, frequency of alcohol intake, leisure time physical activity and body mass index. Fruit and vegetable intakes were also adjusted for each other. (All covariates were included in the models both with and without interaction with time. Age, energy intake and body mass index were centered at the mean – (58yrs, 10.6MJ, 28.5kg/m2))

**Table S7.** Cross-sectional and longitudinal relationship between fruit and vegetable intakes and SBP and DBP, after **further adjustment for Mediterranean diet score**

| Outcome | Food group | Category | **Blood pressure at baseline (mmHg)**  (Cross-sectional association) | | |  | **Blood pressure change per 1-year of follow up (mmHg)**  (Longitudinal association) | | |
| --- | --- | --- | --- | --- | --- | --- | --- | --- | --- |
|  |  |  | **Mean** | (95%CI) | p-value |  | **Mean** | (95%CI) | p-value |
| **SBP** | **FRUIT** | 1st tertile | **143.1** | (140.5, 145.6) | ref. |  | **0.15** | (-0.21, 0.52) | ref. |
|  |  | 2nd tertile | **141.7** | (139.0, 144.4) | 0.019 |  | **0.20** | (-0.17, 0.58) | 0.482 |
|  |  | 3rd tertile | **140.0** | (137.3, 142.7) | <0.001 |  | **0.26** | (-0.12, 0.64) | 0.146 |
|  | **VEGETABLE** | 1st tertile | **143.1** | (140.5, 145.6) | ref. |  | **0.15** | (-0.21, 0.52) | ref. |
|  |  | 2nd tertile | **143.3** | (140.7, 145.8) | 0.740 |  | **0.04** | (-0.33, 0.40) | 0.085 |
|  |  | 3rd tertile | **143.6** | (141.0, 146.2) | 0.345 |  | **0.18** | (-0.19, 0.55) | 0.739 |
| **DBP** | **FRUIT** | 1st tertile | **89.3** | (87.8, 90.7) | ref. |  | **0.14** | (-0.06, 0.35) | ref. |
|  |  | 2nd tertile | **88.8** | (87.3, 90.3) | 0.136 |  | **0.07** | (-0.14, 0.27) | 0.045 |
|  |  | 3rd tertile | **88.0** | (86.5, 89.5) | <0.001 |  | **0.14** | (-0.07, 0.35) | 0.840 |
|  | **VEGETABLE** | 1st tertile | **89.3** | (87.8, 90.7) | ref. |  | **0.14** | (-0.06, 0.35) | ref. |
|  |  | 2nd tertile | **89.4** | (88.0, 90.9) | 0.658 |  | **0.13** | (-0.07, 0.34) | 0.722 |
|  |  | 3rd tertile | **89.3** | (87.9, 90.8) | 0.906 |  | **0.21** | (0.00, 0.41) | 0.129 |

All coefficients were adjusted for age, sex, energy intake, antihypertensive medication, education, marital status, smoking, frequency of alcohol intake, leisure time physical activity and Mediterranean diet score (without the fruit, vegetable and alcohol component). Fruit and vegetable intakes were also adjusted for each other. (All covariates were included in the models both with and without interaction with time. Age, energy intake and Mediterranean diet score were centered at the mean – (58yrs, 10.6MJ, 5.5))

**Table S8.** Cross-sectional and longitudinal relationship between fruit and vegetable intakes and SBP and DBP, **in participants without cardiovascular disease in the medical history**

| Outcome | Food group | Category | **Blood pressure at baseline (mmHg)**  (Cross-sectional association) | | |  | **Blood pressure change per 1-year of follow up (mmHg)**  (Longitudinal association) | | |
| --- | --- | --- | --- | --- | --- | --- | --- | --- | --- |
|  |  |  | **Mean** | (95%CI) | p-value |  | **Mean** | (95%CI) | p-value |
| **SBP** | **FRUIT** | 1st tertile | **143.3** | (140.5, 146.1) | ref. |  | **0.25** | (-0.14, 0.64) | ref. |
|  |  | 2nd tertile | **141.9** | (139.0, 144.8) | 0.023 |  | **0.31** | (-0.09, 0.70) | 0.419 |
|  |  | 3rd tertile | **140.3** | (137.3, 143.3) | <0.001 |  | **0.35** | (-0.05, 0.75) | 0.190 |
|  | **VEGETABLE** | 1st tertile | **143.3** | (140.5, 146.1) | ref. |  | **0.25** | (-0.14, 0.64) | ref. |
|  |  | 2nd tertile | **143.7** | (140.9, 146.5) | 0.549 |  | **0.15** | (-0.24, 0.54) | 0.182 |
|  |  | 3rd tertile | **143.8** | (141.0, 146.7) | 0.446 |  | **0.29** | (-0.10, 0.68) | 0.553 |
| **DBP** | **FRUIT** | 1st tertile | **89.3** | (87.7, 90.9) | ref. |  | **0.15** | (-0.07, 0.36) | ref. |
|  |  | 2nd tertile | **88.9** | (87.3, 90.6) | 0.303 |  | **0.07** | (-0.15, 0.29) | 0.057 |
|  |  | 3rd tertile | **88.3** | (86.6, 90.0) | 0.007 |  | **0.13** | (-0.09, 0.35) | 0.669 |
|  | **VEGETABLE** | 1st tertile | **89.3** | (87.7, 90.9) | ref. |  | **0.15** | (-0.07, 0.36) | ref. |
|  |  | 2nd tertile | **89.7** | (88.1, 91.2) | 0.308 |  | **0.13** | (-0.08, 0.35) | 0.689 |
|  |  | 3rd tertile | **89.4** | (87.7, 91.0) | 0.884 |  | **0.21** | (0.00, 0.43) | 0.107 |

All coefficients were adjusted for age, sex, energy intake, antihypertensive medication, education, marital status, smoking, frequency of alcohol intake and leisure time physical activity. Fruit and vegetable intakes were also adjusted for each other. (All covariates were included in the models both with and without interaction with time. Age and energy intake were centered at the mean – (58yrs, 10.6MJ))

**Figure S1.** Systolic blood pressure trajectories by fruit intake tertiles (separately by 5-year birth cohorts) **in complete case analysis**


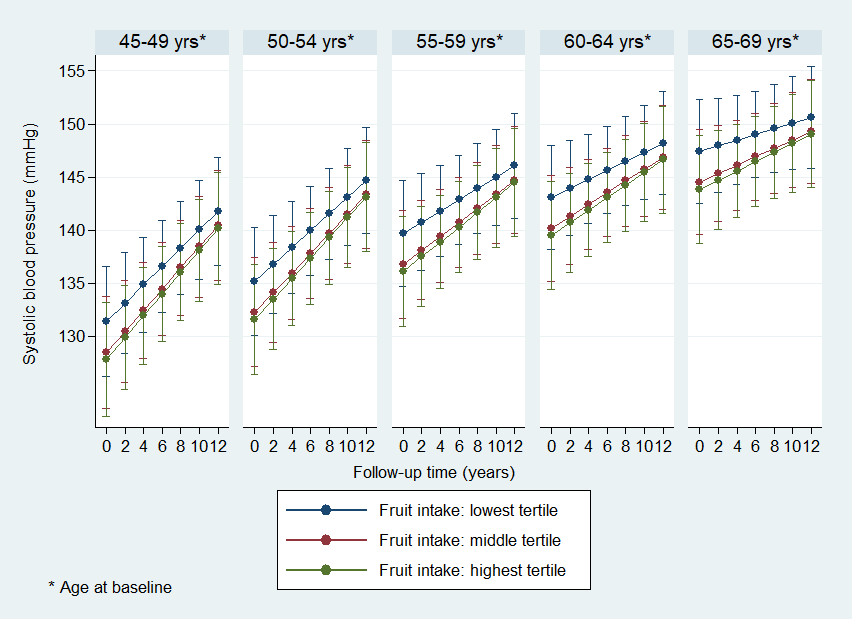


**Figure S2.** Diastolic blood pressure trajectories by fruit intake tertiles (separately by 5-year birth cohorts) in **complete case analysis**


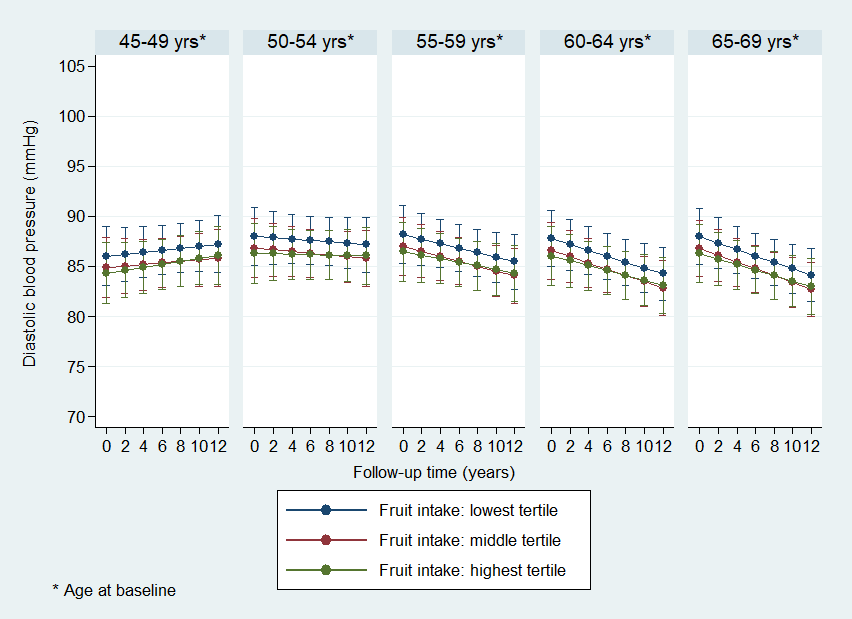


**Figure S3.** Systolic blood pressure trajectories by fruit intake tertiles (separately by 5-year birth cohorts) in **participants who took no antihypertensive medication**


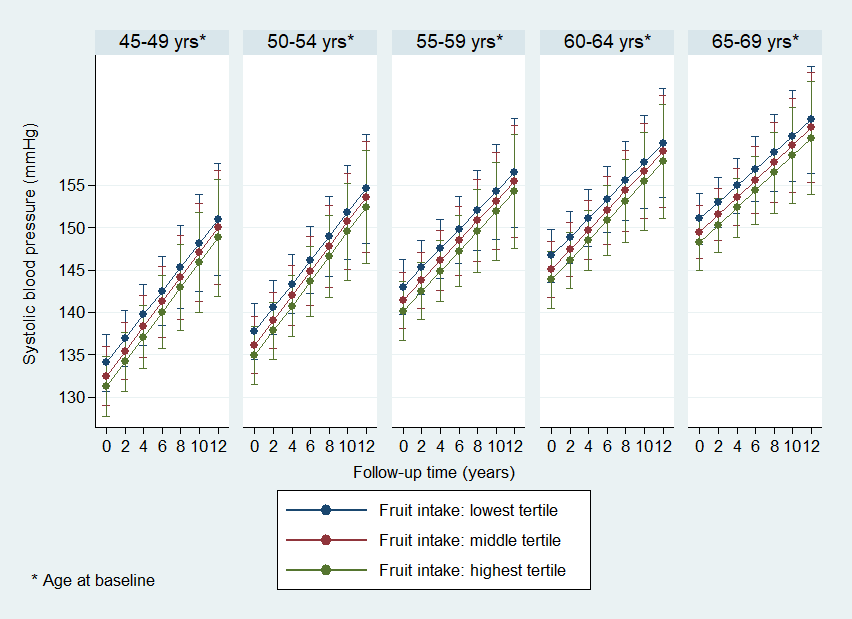


**Figure S4.** Diastolic blood pressure trajectories by fruit intake tertiles (separately by 5-year birth cohorts) in **participants who took no antihypertensive medication**


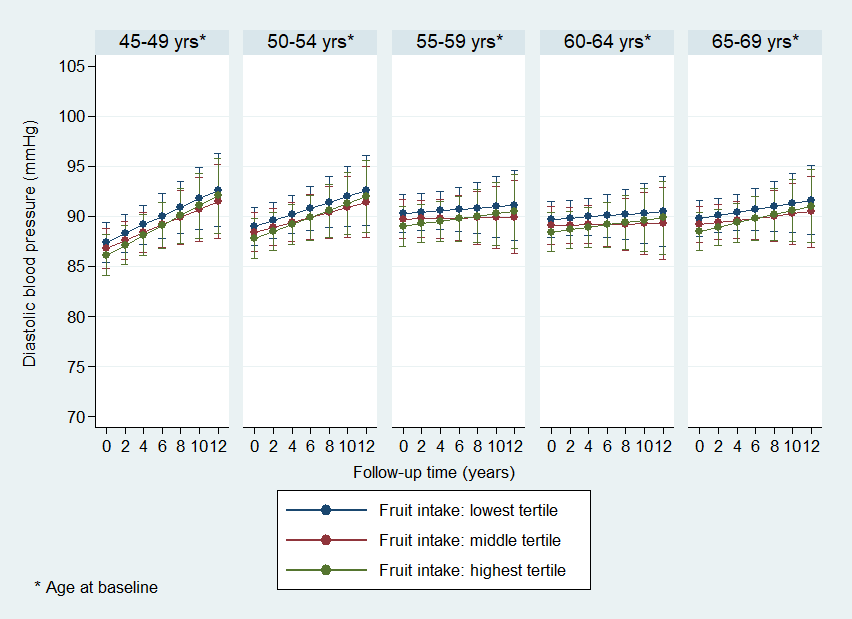

Supplement: Supplementary file 1 — Supplemental material [file 41371_2019_189_MOESM1_ESM.docx]
